# Characteristics of Instrumental Methods to Describe and Assess the Recrystallization Process in Ice Cream Systems

**DOI:** 10.3390/foods8040117

**Published:** 2019-04-04

**Authors:** Anna Kamińska-Dwórznicka, Ewa Gondek, Sylwia Łaba, Ewa Jakubczyk, Katarzyna Samborska

**Affiliations:** 1 Department of Food Engineering and Process Management, Faculty of Food Sciences, Warsaw University of Life Sciences (WULS-SGGW), Nowoursynowska 159C, 02-776 Warsaw, Poland; ewa_gondek@sggw.pl (E.G.); ewa_jakubczyk@sggw.pl (E.J.); katarzyna_samborska@sggw.pl (K.S.); 2 Institute of Environmental Protection-National Research Institute, Krucza 5/11d St., 00-548 Warsaw, Poland; sylwia.laba@ios.gov.pl

**Keywords:** recrystallization, food hydrocolloids, methods for crystal structure evaluation

## Abstract

Methods of testing and describing the recrystallization process in ice cream systems were characterized. The scope of this study included a description of the recrystallization process and a description and comparison of the following methods: microscopy and image analysis, focused beam reflectance measurement (FBRM), oscillation thermo-rheometry (OTR), nuclear magnetic resonance (NMR), splat-cooling assay, and X-ray microtomography (micro-CT). All the methods presented were suitable for characterization of the recrystallization process, although they provide different types of information, and they should be individually matched to the characteristics of the tested product.

## 1. Introduction

The most important factor that determines frozen food quality is the course of crystallization [1]. Crystallization is a process of ice crystal formation as a consequence of atomic ordering and mostly includes hexagonal columns, plates, and dendritic crystal lattices [2,3]. Size, location, and morphology of the ice crystals determine the quality of frozen food, especially ice cream desserts [3,4,5]. Large ice crystals have a negative impact on product textural properties. Ice crystals of sizes between 10 and 20 µm give the product its desired texture whereas ice crystals larger than 50 μm (if present in a significant quantity) cause the product to have an undesirable (coarse or grainy) texture [1,6,7,8,9,10].

**Figure 1:**
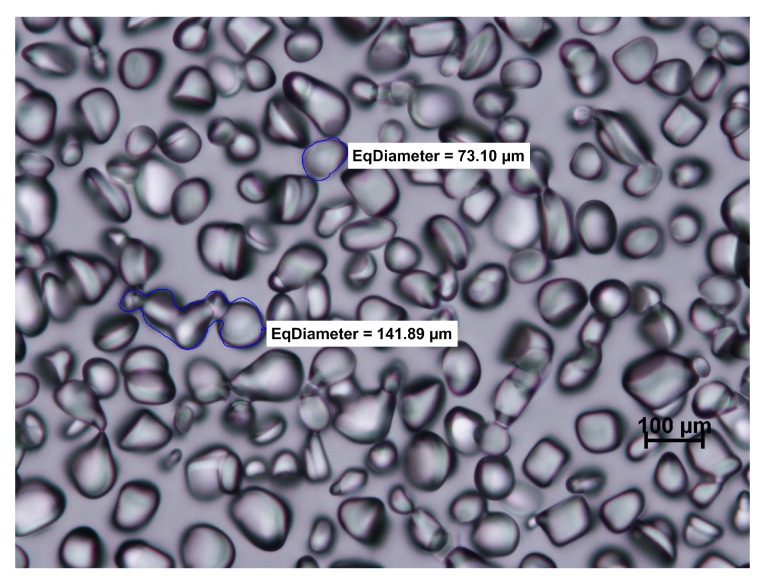
Microscopic image of ice crystals in model sucrose solution (50%) after 96 h of storage at −8 °C; coalescence visible (own work, not published).

Ice cream is a multiphase physicochemical system originating from the dispersion of individual components in different phases [11,12]. The structure of ice cream is formed by dispersion of air in the frozen liquid that consists of approximately two-thirds water. Therefore, ice cream is a foam, a system in which a liquid (dispersing) phase is dispersed in air (dispersed phase). In the water phase, ice cream is a real solution of sucrose, lactose, and other sugars as well as mineral salts, whose particle sizes do not exceed 1 µm [11]. In general, an ice cream system is constituted of four phases [13]: unfrozen matrix (a solution of different mono- and polysaccharides), air bubbles (with sizes between 20 and 150 µm), ice crystals (with sizes from 10 to 75 µm), and fat globules (between 0.4 and 4 µm). Ice formation occurs after initial freezing, accelerates within the first hours after production, and under unstable temperature conditions, during storage, ice crystals grow due to the recrystallization process [8,14]. Various factors, that include total solids, initial freezing temperature, unfrozen water, stabilizer type, sweetener type, and storage temperature influence the excessive crystal growth during storage [14,15]. When temperature fluctuates, unfrozen water diffuses to the surface of existing crystals and enhances their growth (Figure 1).

The recrystallization process occurs at a constant temperature during long storage, especially above the glass transition temperature [16,17,18]. Heat and mass transfer cause some crystals to melt and others to grow [19]. During storage, this ice crystal growth occurs mostly because of two mechanisms, coalescence and migration. Coalescence is the process of gathering two or more adjacent ice crystals that form a kind of bridge between them until a single and much larger ice crystal arises. Migration (Ostwald ripening) consists of two stages: melting of smaller crystals, and movement of melted liquid to the surface of crystals with larger diameters. Water molecules at the surface of small crystals are not firmly bound because of the high curvature. These “free” water molecules tend to diffuse through the freeze-concentrated matrix and are deposited on the surface of the crystals with a larger diameter. The water molecule diffusion process occurs because of the differences in vapor pressure (the vapor pressure is inversely proportional to the ice crystal radius). Usually, these two mechanisms of recrystallization occur simultaneously. Some researchers have claimed that the rate of crystal growth may be dependent on the viscosity of the unfrozen phase [6,8,11,14,20]. However, the influence of selected stabilizers on the recrystallization rate in frozen food systems has been investigated most intensively [1,8,21,22,23].

Hydrocolloid stabilizers are used in food production to modify water-binding capacity, freezing rates, ice crystal formation, and rheological properties [7,8,11,12,24]. Many studies have suggested that some aspects of stabilizer functionality with respect to recrystallization protection may depend on the structure, as measured by rheological properties, which results from the freeze-concentration of the polysaccharide in the unfrozen phase of ice cream. This structure from the stabilizers would affect the rate at which water diffuses to the surface of a growing ice crystal. The stabilizers could also lead to the formation of small curvatures with different radii during ice crystal growth. These newly formed curvatures appear on the surfaces of both smaller and larger ice crystals and prevent differences in vapor pressure between them [1,8,11,21,22,23]. Polysaccharide stabilizers such as guar gum, locust bean gum (LBG), carboxyl methylcellulose, alginate, and xanthan gum are used commonly to control crystal lattice creation.

Different forms of carrageenan are commonly used as stabilizers. The kappa carrageenan form is mostly used to stabilize dairy products, but it may also be applied to control crystal growth in sorbet production [1,25]. The iota fraction of carrageenan reacts electrostatically with milk proteins to form a three-dimensional network that resists separation of the suspended phase in ice cream mixes [11,26]. Gaukel et al. (2014) [8] investigated the impact of a special protein called antifreeze protein (AFP) on the ice recrystallization inhibition process. Due to the fact that recrystallization is a significant problem in frozen food, recrystallization and its inhibition have both been widely studied. Moreover, currently there is interest in the possibilities of applying different methods to describe and control this process during storage as well. Most of the studies related to the measurement of the recrystallization rate consist of determining the ice crystal size distribution and the ice crystal size using microscopy. It is the best known, although not the only method, to describe the recrystallization phenomenon.

The aim of this review was to outline the basic characteristics of the measurement method, sample preparation, and equipment required to show and describe ice crystals during and after the recrystallization process using the following methods: FBRM, OTR, NMR, splat cooling, microscopy analysis, and X-ray microtomography.

## 2. Methods of Testing and Describing the Recrystallization Process

### 2.1. Focused-Beam Reflectance Measurement (FBRM) Technique

Control of the ice crystallization process is mostly conducted in an empirical way, mainly due to a lack of experimental data. Research on ice crystal size distribution (CSD) is not simple, especially because of the possibility of melting under unstable conditions and also when it is about a type of ice cream product that contains three phases [6]. The focused-beam reflectance measurement (FBRM) is a new tool for on-line measurements created to investigate and to monitor CSD during the laboratory and the industrial crystallization processes [6,7,14,27].

**Figure 2:**
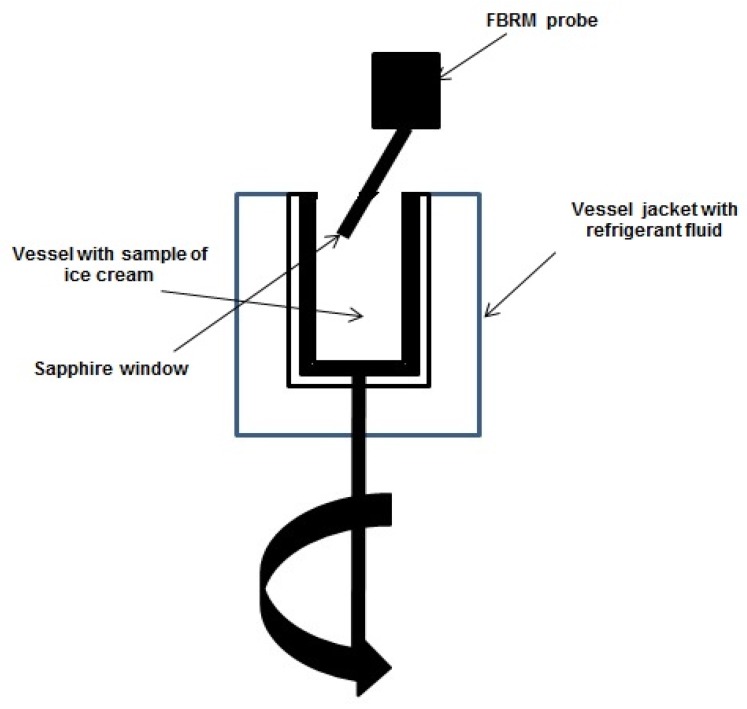
FBRM experimental device (own work, based on Amamou et al., 2010 studies [6]).

The real-time particle size analysis technique of FBRM works by focusing a laser beam directly down the probe tip through a sapphire window. The optical part is rotated about an axis to the probe (2 m/s), so that the beam traces out the circular path (reflected light is detected in the probe). The probe tip is inserted, at an angle, directly into the process streams, to ensure that particles can flow easily across the probe window where the measurement takes place (Figure 2). The laser beam can scan across particle passes near the window. It notes the duration of the reflection and deduces the length of the chord [6,28,29,30].

Generally, the FBRM instrument can acquire thousands of chord lengths per second. On a counter board, these lengths are classified into a series of size ranges that are expressed as a distribution, referred to as a chord length distribution (CLD). This technique considers the shape and dimensions of the particles. However, the CLD value does not gives information about morphology of the particles and it is less useful for the characterization of a crystal lattice in frozen products [6,7,27].

Amamou et al. (2010) [6] presented a study which examined the freezing step that occurs in a scraped-surface heat exchanger during the manufacture of sorbet. The aim of this investigation (using FBRM technique) was to follow the evolution of ice crystals during the freezing of sorbet in the exchanger and to relate this evolution to process parameters. The measurement showed that this method could be used to follow crystal structure in a sorbet consisting of up to 30% ice and that a decrease of temperature during refrigeration accelerates ice crystallization and favors the formation of smaller crystals. They demonstrated that when the initial sucrose concentration in the solution increased, the ice fraction increased more slowly and the mean chord length was smaller.

Arellano et al. (2012) [27] demonstrated, using an example of sorbet freezing, that the FBRM sensor may be a promising tool for monitoring on-line development of ice crystals in a product containing up to 40% ice. Using the FBRM method they proved that an increase of dasher speed slightly decreases chord length of the ice crystals, due to the higher shear of the product, which leads to the attrition of ice crystals, producing new, smaller ice nuclei via secondary nucleation.

The recrystallization process in ice cream using the FBRM technique was investigated by Ndoye and Alvarez (2015) [14]. They compared two commercial and differently stabilized ice creams using an original and real-time particle counting and sizing method. They stored the ice creams for 154 days at four different temperatures (−5, −8, −12 and −18 °C) and three amplitudes of temperature fluctuations (±0.1, ±0.75, ±2.5 °C). The crystal size distributions (CSD) were assessed at various time intervals and the recrystallization kinetic data were obtained by fitting the experimental results to the asymptotic Ostwald ripening model. As they expected, recrystallization rates increased with mean storage temperature and amplitude of temperature fluctuations. In the samples of ice cream, they compared which of the stabilizing systems worked better, concluding that the carrageenan seemed to be more effective than LBG. For both ice cream samples, it was proven that ice crystal size increased as a function of time.

The main advantage of the FBRM technique seems to be its suitability for on-line measurements of high solid-concentration suspensions and for following rapid crystallization kinetics. However, it only provides information about the total number of ice crystals and the changes of diameters of ice crystals, and therefore the shape and the changes of ice crystal location are measured.

### 2.2. Oscillatory Thermo-Rheometry Technique (OTR)

It was shown that the dynamics of rheological measurements can be used for characterization of textural properties and structure of foamed dairy emulsion. The dynamic storage modulus G’ (elastic response) and loss modulus G” (viscous behavior) can provide information about properties of viscoelastic materials.

Stanley et al. (1996) [31] proved that the modulus (G’) greatly increases when the ice cream temperature decreases, hence increasing the ice fraction in ice cream. Smith et al. (2000) demonstrated that microstructure in whipped cream influenced the dynamics of oscillatory storage (G’) and loss (G”) moduli. The parameters both decreased with a coarser foam structure due to increased air bubble sizes during storage. This method, called oscillatory thermo-rheometry (OTR), allows one to distinguish whether the recrystallization process occurred, but does not provide information about the sizes of ice crystals or the changes in shape and location. That kind of investigation is usually complemented by microscopic analysis or the FBRM technique.

Wildmoser et al. (2004) [32] used rheology for the microstructural and sensorial assessment of ice cream samples, produced with application of different ice cream mix compositions and processes. Rheological properties of ice cream were examined in a rotational rheometer (plate-plate geometry). The creaminess and other sensory factors were investigated in order to correlate them with the results gained in the rheometer. This tool was used to perform oscillatory measurements at low deformation amplitudes for three different temperature ranges to assess the rigidity and “scoopability” of ice cream at a low temperature from −10 to −20 °C. The higher the overrun and the smaller the connectivity of ice crystals were, the smaller were the measured values of moduli G’ and G”. In this study, the OTR technique was accomplished using a cryo-scanning electron microscopy (cryo-SEM) to investigate the ice crystals and the air bubble sizes. As the degree of connectivity of ice crystals increased, the storage and loss moduli at temperatures below −10 °C increased. In the temperature range above 0 °C, air and fat phases played a major role in the rheological behavior, due to the total ice crystal melting processes. The loss modulus G” increased by a factor of approximately 10 when the air content was increased from 0 to 100%.

Eisner et al. (2005) [33] examined the microstructure of ice cream made using a relatively low viscosity vanilla ice cream mixture, prepared in a freezer with outlet temperature of approximately −5 °C and stored for 2 weeks at −25 °C. Using the OTR method and cryo-scanning electron microscopy (LT-SEM) they found that ice cream foam stability correlated with the sensed creaminess and could be improved with smaller air cells and reduced coalescence. At the temperature range from −20 to −10 °C the microstructure of ice crystals was dominant and the storage modulus G’ decreased while the loss modulus G” showed a plateau, correlated with the rigidity and “scoopability” of the ice cream. For temperatures from −10 to 0 °C the ice fraction decreased significantly. At temperatures above 0 °C both moduli (G’ and G”) showed a lower plateau, which correlated with the sense of creaminess. Microstructural study may improve fat agglomeration with further enhancement of foam stability, and it correlates with reduced ice crystal sizes.

Sensory quality closely correlates with viscoelastic properties of products. Tsevdou et al. (2015) [34] correlated vanilla ice cream sensory characteristics with the changes in rheological behavior (using the OTR method), during storage under static and dynamic temperature conditions. The formation of ice crystals was estimated in terms of mouthfeel perception. For temperatures in the range of −30 to −5 °C changes in the value of G’ (loss modulus) were observed over a certain period of time, suggesting that the recrystallization phenomenon is not only time, but also temperature dependent. At high storage temperatures such as −5 °C, the G’ value showed that ice recrystallization occurred to a greater extent than at temperatures below −12 °C. It was found that viscoelastic properties correlated with sensory perception for ice crystal formation during storage at isothermal temperature conditions and temperature fluctuations, and thus could be used to predict the quality and the remaining shelf-life of ice cream without recrystallization changes. However, the OTR method did not provide information about changes in shapes and diameters of ice crystals, and therefore it cannot give a precise evaluation of the recrystallization process.

### 2.3. Nuclear Magnetic Resonance (NMR) Technique

Content, purity, and molecular structure of a sample can be determined using an analytical chemistry technique called nuclear magnetic resonance (NMR). When the NMR technique is used it is possible to quantitatively analyze mixtures containing known compounds. For unknown compounds, NMR can either be used to match against spectral libraries or to infer the basic structure directly. The NMR technique can be used to determine molecular conformation in solution and to study physical properties at the molecular level such as conformational exchange, phase changes, solubility, and diffusion. In order to achieve the desired results a variety of NMR techniques are available. In addition, NMR is versatile and has the potential to be nondestructive, which makes it a potential tool in quality control of various products, including milk-based desserts [35].

The principle of the method relates to the fact that many nuclei possess spin and all nuclei are electrically charged. If an external magnetic field is applied, energy transfer is possible from the base energy to a higher energy level (generally a single energy gap). The energy transfer takes place at a wavelength that corresponds to the radiofrequencies, and when the spin returns to its base level, energy is emitted at the same frequency. The signal that matches this transfer is measured in many ways and processed in order to obtain an NMR spectrum for the charged nucleus [36,37].

The NMR technique has been shown to be an appropriate method to calculate the amount of unfrozen water in a food sample. Lucas et al. (2004) [38] examined liquid from the solid water in aqueous sucrose solutions (sucrose and/or casein). They considered spin-spin relaxation measurements that were usually used and also spin-lattice ones. They showed that spin-lattice relaxation provides information about the ice molecular structure. This work confirmed that the ice phase in the case of sucrose solutions is composed of pure water.

Hagiwara et al. (2006) [39] investigated the relationship between the recrystallization rate of ice crystals in sugar solutions (sucrose, maltose, glucose and fructose) and the water mobility in a freeze-concentrated matrix. They observed ice crystals during the recrystallization process using the cryomicroscope system. Their study was complemented with an NMR study in order to examine water mobility via the self-diffusion coefficient of the water component. They found that the recrystallization rate in a variety of sugar solutions depended significantly on the water mobility in the freeze-concentrated matrix and that the self-diffusion coefficient of the water component was a useful parameter to predict and control the recrystallization rate. Brown et al. (2014) [40] used NMR relaxation and time-dependent self-diffusion measurements to monitor the three-dimensional changes to the vein network in ices with and without addition of the ice binding proteins (IBP called antifreeze protein and AFP). They found that the NMR technique was useful in evaluation of the impact of IBPs (among other things) on the vein network structure and the recrystallization process. The IBPs were found useful to inhibit recrystallization and to modify the three-dimensional ice structures, resulting in persistent small size of ice crystals and shorter diffusion of distances along the vein.

It has been discovered that NMR could be used to determine fat globule size in ice cream and to determine the effect of the formulation on hard ice creams’ structure [36,41,42]. Lucas et al. (2005) [43] presented the NMR technique as a nondestructive method to characterize the behavior of both fat and water in ice cream mixtures in the frozen state. They proved that the NMR technique described the crystallized and liquid phases separately, and that they could be applied to determine the amount of unfreezable water and mobility of the freeze-concentrated phase. The NMR technique was also used to determine the impact of the quantity of crystals and their organization on the mechanical properties and textures of ice cream mixes [35,43].

The NMR technique does not involve any thermal processes to assess the amount of ice and thus can be performed at a stable temperature. It is also important that the relaxation parameters of water were used to provide information on the water/non-water molecule interactions. This technique may be a valuable tool for understanding how various stabilizers affect the three-dimensional vein network and recrystallization processes.

### 2.4. Splat-Cooling Assay

In order to determine the ice recrystallization inhibition (IRI) activity of ice cream stabilizers, the capillary method or splat-cooling assay can be performed [44]. Although it is an older method, it is a simple and valuable capillary method for studying recrystallization inhibition which is usually based on loading the samples into 10 µL glass capillaries (51 mm long, 1 mm outer diameter) by capillary action. Capillaries containing dilution series (for example different AFP concentrations to determine at what level that IRI activity is lost) are later folded together exactly, with no space between adjacent capillaries. Subsequently, all the series were snap frozen in different organic compounds, for example, 2,2,4-trimethylpentane or 95% ethanol and cooled to approximately −60°C with dry ice. After the snap freezing, samples were immersed in a jacketed beaker filled with 50% ethylene glycol in order to maintain the temperature at the level of −6 °C. The incubation took 16 to 20 h and later microscopy and image capture were done using polarizing light filters [45,46]. These methods used a simple set-up and allowed the analysis of the IRI activity for a series of samples within one field of view. Tomczak et al. (2003) [45] claimed that the capillary method allowed samples to be aligned and viewed simultaneously, which facilitated the determination of the IRI endpoint. They noted that after the samples have been prepared they could be archived in a freezer for future IRI activity analysis. In fact, sample preparation was not so easy and could be problematic.

The IRI activity of different substances such as AFP and some polysaccharides can be demonstrated using “splat” assay. This is not a new method and has been used for nearly twenty years. In these experiments, a small volume of sample was usually expelled from a height of 1.5 to 3 m onto a metal plate (for example an aluminum block) that had been cooled in liquid nitrogen or dry ice. The sample drop froze upon hitting the metal plate, forming a thin (splat) wafer of ice. This wafer was later transferred to a microscope stage at a high sub-zero temperature where the sample recrystallized over time and annealing temperature (for example: −10, −8, −6, −4 °C). Different modifications of this method are possible, for example, the sample could be snap frozen between two cover slips. This technique was complemented by crossed polarizers with a dissecting microscope and ice-binding activities of some substances that were examined by Raymond and Knight (2003) [47]. The splat cooling technique had the advantage that ice crystal growth was directly observed and hence it was easier to interpret. However, preparation of each individual coverslip was problematic, and in addition must be photographed separately and only after the photographs or images are assembled and analyzed [45,46,48].

### 2.5. Microscopy and Image Analysis

The most popular and universal method of testing and describing the recrystallization process is direct light microscopy observation followed by image analysis of the ice crystals. When the work was started with another technique such as oscillatory thermos-rheometry or splat cooling assay, to complete it the work was usually supported by an appropriate microscopy technique, a cryo-scanning electron microscope or a microscope with a polarizer [32,44]. Physicists studying polar ice structures proposed examining the structures by the method of direct observation using an optical microscopy with episcopic coaxial lighting [49], and it was later adapted by Faydi et al. (2001) [50]. Caillet et al. (2003) [51] recommended the direct microscopy method as a good technique for analysis of frozen food structure. They compared it with two other methods: a destructive method by dispersion and observation by light microscopy; and an indirect method, by scanning electron microscopy after freeze-drying the sample. The three methods examined led to the same conclusions for the examination under the same conditions of freezing.

Donhowe and Hartel (1996a, 1996b) [16,17] examined sizes of ice crystals in ice cream with an optical microscope placed in a refrigerated glove box. Samples of ice cream were stored at −14 °C for several hours. Photomicrographs of ice crystals were taken within 15 min of the sample preparation (no change in ice crystal size occurred over the time period of measurement). Negatives were enlarged and analyzed. This assay led to the conclusion that recrystallization in ice cream stored in bulk containers increased with mean storage temperature for both constant and varying temperatures. Ice crystal sizes in these samples increased linearly with time as well.

Regand and Goff (2003) [12] presented a study of stabilized ice cream model systems. Small drops of different solutions with the addition of some hydrocolloid stabilizers were placed between a slide and cover slips, frozen to −50 °C, then cycled between −3.5 °C and −6 °C on a cold stage of the light microscope, and then the images were acquired using a camera. The ice crystals were later counted and measured individually from the images (at least 200 crystals for the sample). They based the conclusion on a logistic model of ice crystal size distributions characterized earlier by Flores and Goff (1999a, 1999b) [52,53]. This method allowed them to obtain the ice crystal diameter at 50% of cumulative distribution of the sample (X_50_) and the slope of cumulative distribution at X_50_. The recrystallization rate was calculated as the slope of linear regression of the curve plotted with values of X_50_ for each cycle. Using this method, they proved there was significant retardation of recrystallization with the addition of sodium alginate and xanthan. The same technique (using different equipment and a different image analysis program) was first used to explain the IRI activity of kappa carrageenan hydrolysates in model sucrose solutions [4] (Table 1) and later in ice cream sorbet [1] (Table 2).

**Figure 3:**
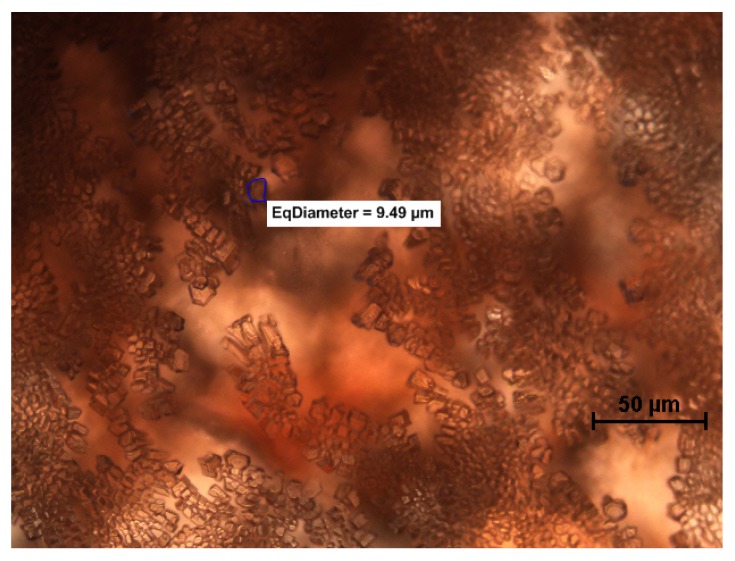
Microscopic image of ice crystals in strawberry sorbet with the addition of AFP III (0.000002%) after one month of storage at −18 °C (own work, not published).

The method described above makes it possible to analyze not only the changes in diameter of ice crystals but also their shape and location. On the basis of the images it is possible to analyze the mechanism of recrystallization. From the shape of the ice crystal, it is sometimes easy to read that accretion between adjacent crystals occurred (Figure 1). Numerous studies have shown that the shape of the ice crystal is influenced by the temperature cycle and by the addition of active substances. Gaukel et al. (2014) [8] focused on crystal morphology in model sucrose solutions with the addition of different types of AFPs. They constructed their theory based on microscope and image analysis. On the basis of the shape of the ice crystals, they claimed that the kappa carrageenan molecule interacted with the ice crystal surface similar to the AFP interactions. The AFPs (also called IBPs) were identified in the blood of Antarctic fish, and then they were found in different organisms. It was discovered [54] that by adsorption of AFP to the ice crystal surface, ice growth was only possible between proteins, leading to a micro curvature. The exact IRI mechanism of AFP is still not fully understood. In the case of pure sucrose solution (Figure 1), typical round ice crystals were formed. In contrast (Figure 3), the shape of ice crystals in sucrose with AFP III present was angular, elongated, and gave the illusion of a three-dimensional structure.

**Figure 4:**
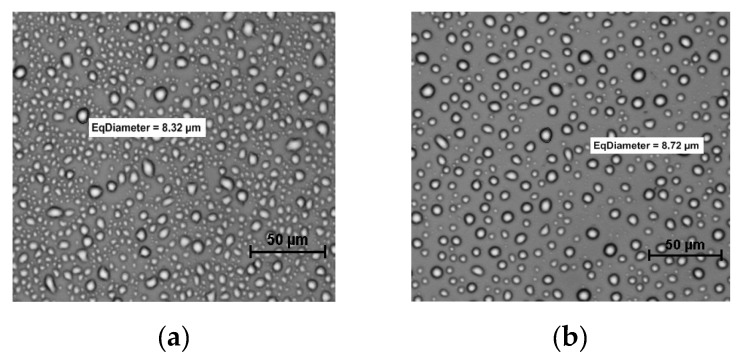
Microscopic images of ice crystals in model sucrose solutions with the addition of enzymatic hydrolysates of κ-carrageenan (after HCL hydrolysis), after 24 h (**a**), and 96 h (**b**) of storage at −8 °C (Kamińska-Dwórznicka et al., 2016 [5]).

When the growth rate of ice crystals in samples with the IRI active substances is small, there is no difference in morphology or size of ice crystals, as was observed by Kamińska-Dwórznicka et al. (2016) [5] for kappa carrageenan hydrolysates added to model sucrose solutions (Figure 4).

### 2.6. X-ray Microtomography (Micro-CT)

**Figure 5:**
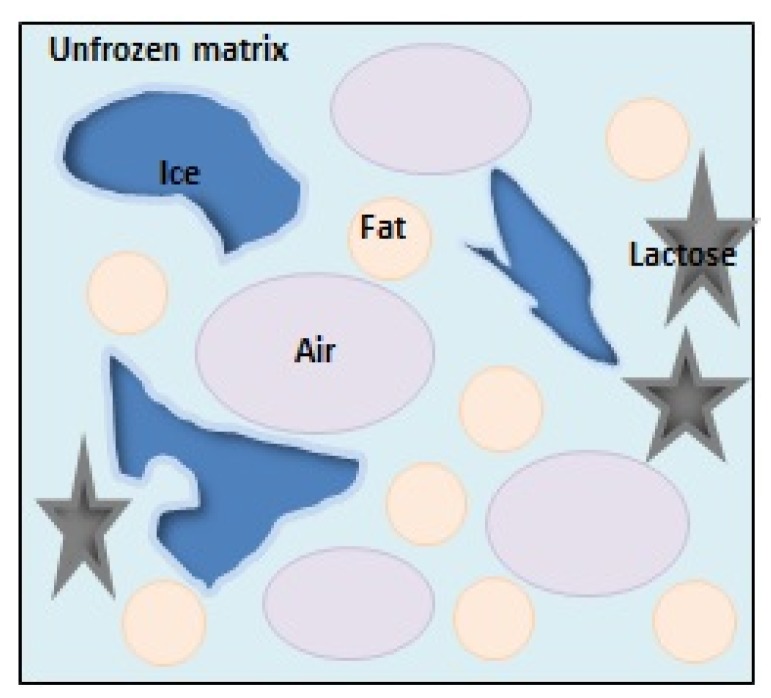
Schematic diagram showing the complex microstructure of ice cream visible during micro-CT analysis (own work, based on Guo et al., 2017 [57]).

The technique of microscopy and image analysis has brought positive results in ice crystal microstructure investigations. However, it is a two-dimensional technique and it does not characterize multidimensional structure of ice cream mix before and after freezing. During the recrystallization process, the microstructure of ice cream (Figure 5) changed (not only in ice crystal size but also in size of fat globules and air bubbles). It was possible to overcome these (and other limitations) with the X-ray microtomography (micro-CT) three-dimensional technique [55,56].

The micro-CT method is based on the same assumptions as classic tomography. However, by using a smaller radiation spot, it is possible to obtain a higher resolution of the reconstructed image [55,58]. This non-destructive method was used by Pinzer et al. (2012) [13] to examine the three-dimensional distribution of the three main phases in ice cream model systems. A cylindrical sample holder (10 mm diameter) was filled with a piece of ice cream and placed inside the CT scanner which was programmed to start a scan every 4 h. The cold laboratory was programmed to follow a temperature step function, varying between −20 and −8 °C. When analyzing only the process of recrystallization, they found that during cold periods elongated ice crystals were formed. Because of the temperature fluctuation, partial melting occurred and elongated ice crystals split up again into smaller ones. They concluded that a partial melting–refreezing mechanism was the dominant coarsening mechanism for the investigated storage conditions. Hence, both the size and structure of ice crystals, as well as the mechanisms of those microstructural changes were examined. However, they encountered some difficulties in the interpretation of images caused by the limited resolution of desktop microtomographs and the poor contrast between different phases, which introduced systematic errors.

Guo et al. (2017) [57] presented results for micro-CT measurements of thermal changes in the microstructure of ice creams after 0, 7 and 14 cycles at temperatures ranging between −15 and −5 °C. For tomography, 3 mm diameter tubes were filled with ice creams just before the analysis (a bed of dry ice was used to avoid any changes). With respect to the recrystallization process, they found that melting and solidification had the greatest impact on the final ice cream microstructure. Temperature conditions during the first seven thermal cycles promoted migratory recrystallization. The sizes of the ice crystals increased while the number of the ice crystals decreased. However, the growth rate of crystals after the seven cycles decreased significantly, which was related to the limited amount of available water from the unfrozen matrix, adjacent ice crystals, and air cells.

## 3. Conclusions

The technique of microscopy and image analysis allows one to describe ice crystal microstructure. From the images of the ice crystals we can easily obtain information about the size and the changes of their shapes and location during storage at different temperature and time conditions. The images can be easily analyzed using specific computer software. The main disadvantages of this method are difficulties in the preparation of samples and its influence on the repeatability of results. The technique of X-ray microtomography seems to offer a new possibility in the analysis of the recrystallization process as a non-destructive method that shows ice cream samples in 3D, but has some difficulties with the final interpretation of images. The FBRM (focused beam reflectance) technique is fully automated and provides results more easily and faster than simple image microscopy and image analysis. It is a suitable method for in situ measurements, and it allows sample preparation to be avoided because the measurement is conducted by the probe immersed in the ice cream mixture. However, it can only provide information about changes in the diameters of crystals, without shape and location analysis. The OTR (oscillatory thermo-rheometry) technique is a method in which viscoelastic properties of ice cream closely correlate with the sensory quality, and hence it provides information about the shelf-life of ice cream without recrystallization changes. However, the changes in size or shapes and location of ice crystals are not measured. Nuclear magnetic resonance (NMR) is an effective method to evaluate the amount of unfrozen water in a food sample. Hence, it is a valuable tool for understanding the impact of a stabilizer on the recrystallization processes without providing any information about sizes of ice crystals and locations. Splat-cooling assay is the oldest method to describe the amount, sizes, and morphology of ice crystals. It is a method with a very specific technology for sample preparation and is not suitable for different types of frozen food.

All of the discussed methods are suitable for describing the recrystallization processes, although they provide different types of information, and they should be matched individually to the characteristics of the tested product.

## Figures and Tables

**Table 1:** X_50_ parameter value for model sucrose solutions with the addition of kappa carrageenan and its hydrolysates (value estimated from data presented by Kamińska-Dwórznicka et al., 2015 [4]).

Sample	X_50_ (after 24 h) (µm)	X_50_ (after 96 h) (µm)
30% suc + KK	6	20
30% suc + 3 h HCL	7	8
30% suc + 1.5 h H_2_SO_4_	7	12

Explanatory notes: suc: sucrose solutions, KK: kappa carrageenan, 3 h HCL: hydrolysates after 3 h of hydrolysis in HCL acid, 1.5 h H_2_SO_4_: hydrolysates after 1.5 h of hydrolysis in H_2_SO_4_ acid.

**Table 2:** X_50_ parameter value for strawberry sorbet with the addition of kappa carrageenan and its hydrolysates (value estimated from data presented by Kamińska-Dwórznicka et al., 2015 [1]).

Sample	X_50_ (after 24 h) (µm)	X_50_ (after 96 h) (µm)
sorbet + KK	6	20
sorbet + 3 h HCL	7	8
sorbet +1.5 h H_2_SO_4_	7	12

Explanatory notes: KK: kappa carrageenan, 3 h HCL: hydrolysates after 3 h of hydrolysis in HCL acid, 1.5 h H_2_SO_4_: hydrolysates after 1.5 h of hydrolysis in H_2_SO_4_ acid.
